# Supplementary figures and images for: Divergent hepaciviruses, delta-like viruses, and a chu-like virus in Australian marsupial carnivores (dasyurids)
Source: Virus Evol. 2023 Oct 14;9(2):vead061. doi: 10.1093/ve/vead061 (PMC10630069; doi:10.1093/ve/vead061)

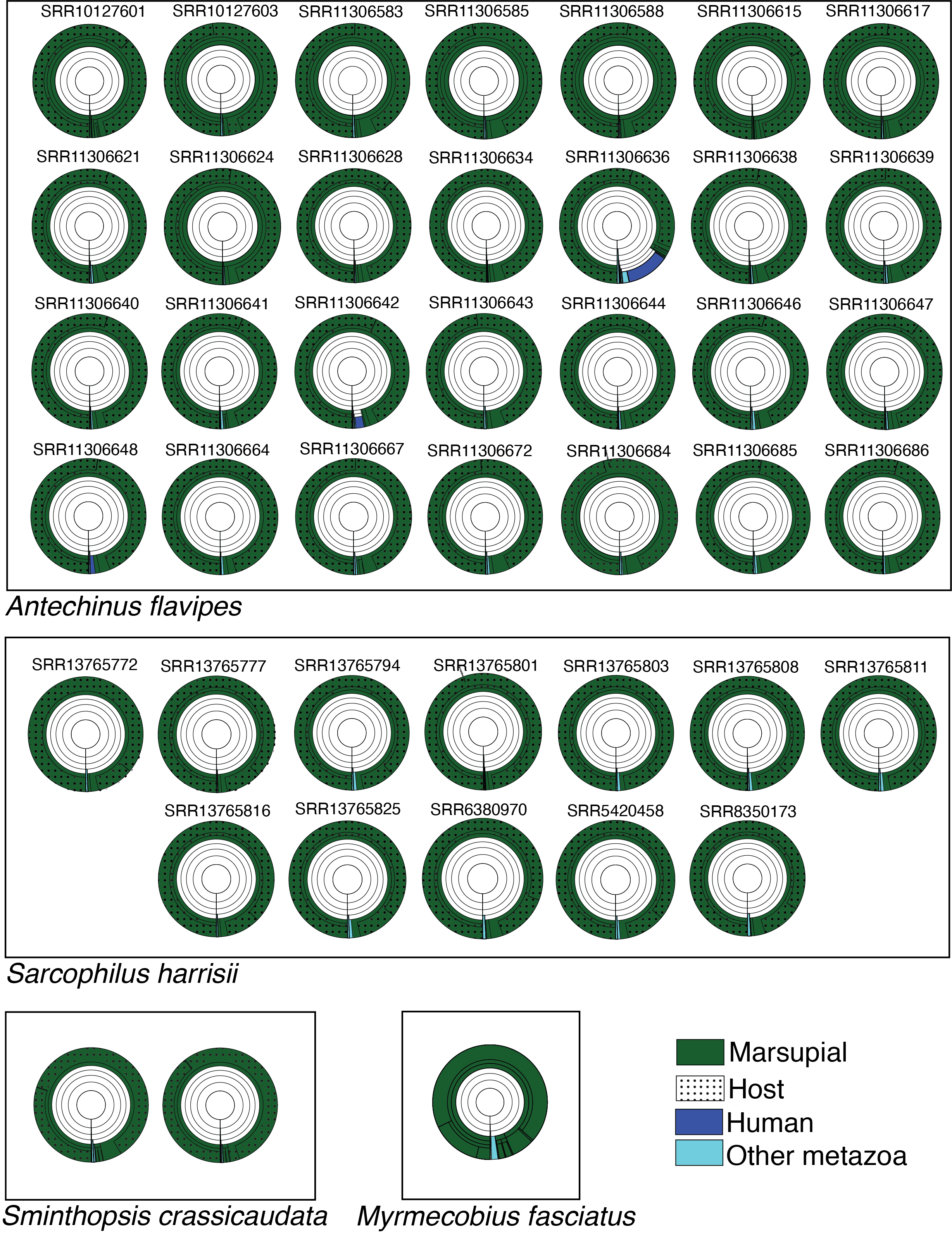

Supplement: vead061_Supp [file vead061_supp.zip › Supplementary figure 1_ccmetagen_Krona_plots.png]
